# Propolis potentiates the effect of cranberry (*Vaccinium macrocarpon*) against the virulence of uropathogenic *Escherichia coli*

**DOI:** 10.1038/s41598-018-29082-6

**Published:** 2018-07-16

**Authors:** Jérémy Ranfaing, Catherine Dunyach-Remy, Laurence Louis, Jean-Philippe Lavigne, Albert Sotto

**Affiliations:** 1 French National Institute of Health and Medical Research, Unit 1047, University Montpellier, Faculty of Medicine, Nîmes, 30908 France; 2 Department of Microbiology, Nîmes University Hospital, Nîmes, 30029 France; 3 Plateforme Génomique et transcriptomique, UMR_S 910, University Aix-Marseille, Faculty of Medicine La Timone, Marseille, 13385 France; 4 Department of Infectious Diseases, Nîmes University Hospital, Nîmes, 30029 France

## Abstract

Uropathogenic *Escherichia coli* (UPEC), the most prevalent bacteria isolated in urinary tract infections (UTI), is now frequently resistant to antibiotics used to treat this pathology. The antibacterial properties of cranberry and propolis could reduce the frequency of UTIs and thus the use of antibiotics, helping in the fight against the emergence of antibiotic resistance. Transcriptomic profiles of a clinical UPEC strain exposed to cranberry proanthocyanidins alone (190 µg/mL), propolis alone (102.4 µg/mL) and a combination of both were determined. Cranberry alone, but more so cranberry + propolis combined, modified the expression of genes involved in different essential pathways: down-expression of genes involved in adhesion, motility, and biofilm formation, and up-regulation of genes involved in iron metabolism and stress response. Phenotypic assays confirmed the decrease of motility (swarming and swimming) and biofilm formation (early formation and formed biofilm). This study showed for the first time that propolis potentiated the effect of cranberry proanthocyanidins on adhesion, motility, biofilm formation, iron metabolism and stress response of UPEC. Cranberry + propolis treatment could represent an interesting new strategy to prevent recurrent UTI.

## Introduction

Urinary Tract Infections (UTIs) are the most common bacterial infections^1^, affecting nine million women in America and 150 million people worldwide each year^2,3^. Women are predominantly affected by UTIs, with 50% of women presenting an UTI during their life, of which 25% will develop a recurrent UTI^2,4^. Uropathogenic *Escherichia coli* (UPEC) are the major pathogen involved in UTIs^5^. Over recent years, these bacteria have developed mechanisms of resistance against β-lactams, cotrimoxazole and fluoroquinolones, the antimicrobial agents usually used in these infections^4,6^. The incidence of multidrug resistant *E. coli* has dramatically increased since the beginning of the century^7^. In this context, it is essential to develop new strategies to prevent or treat UTIs.

Recent evidence suggests that cranberry is effective at preventing UTIs^8–10^, largely due to its anti-adherence properties. The A-type proanthocyanidins (PAC-A) in cranberry have been shown to be important inhibitors of Type-I fimbriae *E. coli* adhesion to uroepithelial cells by modifying the bacteria form, inducing cell rounding and thus reducing its surface of adherence^11,12^. Propolis, a resinous material produced by bees from mixing plant materials with wax and bee enzymes^13^, has been long utilized for its antiseptic and local anesthetic properties. It also displays antimicrobial, anti-inflammatory, anti-tumour, immuno-modulatory and anti-oxidant activities, among others^14^. A previous study showed that propolis could amplify the impact of PACs, offering some protection against UPEC anti-adhesion activity, bacterial multiplication and virulence^15^. Moreover a recent study showed that cranberry and propolis supplementation involved a significant reduction of the incidence of UTIs^16^. The objective of this study was to evaluate the impact of cranberry PAC, propolis and a combination of these two components on the transcriptome of UPEC.

## Results

### Cranberry and propolis affect UPEC virulence

**Figure 1:**
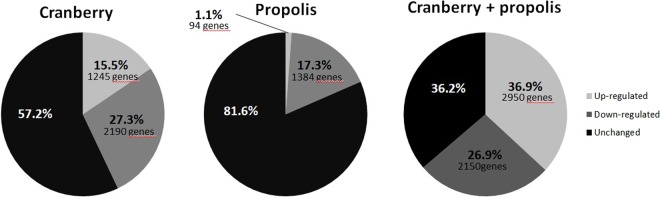
Microarray results. Proportion of genes up-regulated and down-regulated by cranberry (190 μg/mL) and propolis (102.4 μg/mL) alone and combined. Cranberry up-regulates 1,245 genes and down-regulates 2,190 genes. Propolis up-regulates 94 genes and down- regulates 1,384 genes. Cranberry + propolis up-regulate 2,950 genes and down-regulate 2,150 genes.

Expression levels of the entire genome of a clinical *E. coli* strain were measured in the presence of the cranberry and propolis products separately and compared to those of the untreated isolate. The expression level of 5,379 open reading frames was determined. Their log relative transcription levels are shown in Table S1. Overall, 1,245 and 94 genes were found to be up-regulated and 2,190 and 1,384 were found to be down-regulated by cranberry and propolis alone, respectively (Fig. 1).

#### Genes involved in adhesion

As previously observed^15,17–20^, cranberry inhibited UPEC adhesion. Thus, genes encoding Type-1 (*fimBFGHI*) and Type-P fimbriae (*papCDEGHIK*) were significantly down-expressed. Moreover similar down-regulation was observed with genes encoding chaperone usher fimbriae (*yad*, *ycbQ*) and biogenesis of type IV pili (*hofBCQ*). Interestingly, propolis alone also acts on other genes involved in UPEC adhesion: a significant down-expression of genes involved in fimbriae synthesis (*fimZ*, *papA*) and a conserved adhesin (*ecpD*) was noted.

#### Genes involved in motility pathway

Cranberry exposure caused significant down-regulation of genes involved in flagellar biosynthesis (*fliHJPTGLSMOFS*, *flgAHK*), components of motor (*mot*AB) and in flagellar assembly (*div*). Moreover, cranberry treatment also resulted in over-expression of a repressor gene linked to motility (*nsrR*). Similarly, propolis exposure caused down-regulation of a gene involved in filament structure (*fli*C).

#### Genes involved in iron metabolism

As previously observed^21,22^, cranberry treatment caused an up-regulation of genes encoding iron metabolism regulators (*fur*), transport protein (*feo*AB), genes involved in biogenesis pathway of Fe-S cluster (*iscAS*) and iron storage (*ftn*A, *acnA, sodB*). Propolis treatment alone had no significant effect on this pathway.

#### Genes involved in stress response

Cranberry treatment caused significant over-expression of genes involved in stress response (*arc*A, *rpo*S, *cpx*R), periplasmic stress (*rseAC*) and oxidative stress (*oxyR*). Moreover, propolis resulted in overexpression of a gene involved in membrane stress (*psp*A) and in oxidative stress (*csiD*).

#### Genes involved in biofilm formation

Cranberry treatment resulted in significant down-expression of genes involved in the production of exopolysaccharide (*yjb*HF) and chemotaxis (*tsr*). Propolis also caused down-regulation of a gene important for biofilm maintenance (*emr*E).

### Propolis potentiates the effect of cranberry

Microarrays were then performed for the same clinical *E. coli* strain in the presence of both products. Their log relative transcription levels of the combined treatment are shown in Table S1. Upon exposure to cranberry + propolis, 2,950 genes were found to be up-regulated and 2,150 were found to be down-regulated (Fig. 1). In large part, the same genes expression results were obtained for the combination as for cranberry or propolis alone, however some other modifications were observed in the combined treatment, with a notably increased number of up-regulated genes, suggesting a potentialisation of cranberry effect by propolis.

#### Genes involved in adhesion

The combination of cranberry + propolis had an impact on genes with anti-adhesion activity. Thus, genes encoding Type-1 and Type-P fimbriae (*fimC*, *papF*), and curli filament (*csgB*) were significantly down-expressed.

#### Genes involved in motility pathway

The combination of cranberry + propolis caused a down-expression of genes involved in flagellar biosynthesis (*fli*AR) and up-regulation of a key regulator (*lrhA*), thus increasing the loss of UPEC motility.

#### Genes involved in iron metabolism

The iron metabolism induced by cranberry alone was also increased in the combined treatment with a significant up-expression of genes encoding iron transport (*efe*B, *fep*A), iron storage (*ftn*B) and an activator of iron transport (*fea*R).

#### Genes involved in stress response

Cranberry + propolis caused an up-regulation of genes involved in oxidative stress (*ygg*E), and membrane stress (*rse*B, *spy* and *usp*B).

#### Genes involved in biofilm formation

Cranberry + propolis resulted in a down-expression of genes involved in the production of exopolysaccharide (*bsc*C), the initiation of biofilm (*tqs*A), biofilm maintenance (*bdm*) and chemotaxis (*che*AR, *mal*E).

### Confirmation of gene expression changes by qRT-PCR

In order to confirm results obtained by microarray analysis, we determined the expression of randomly selected genes from each metabolic pathway (adhesion, motility, iron metabolism, stress response, and biofilm formation) and for each condition (cranberry, propolis and cranberry + propolis) using qRT-PCR.

**Figure 2:**
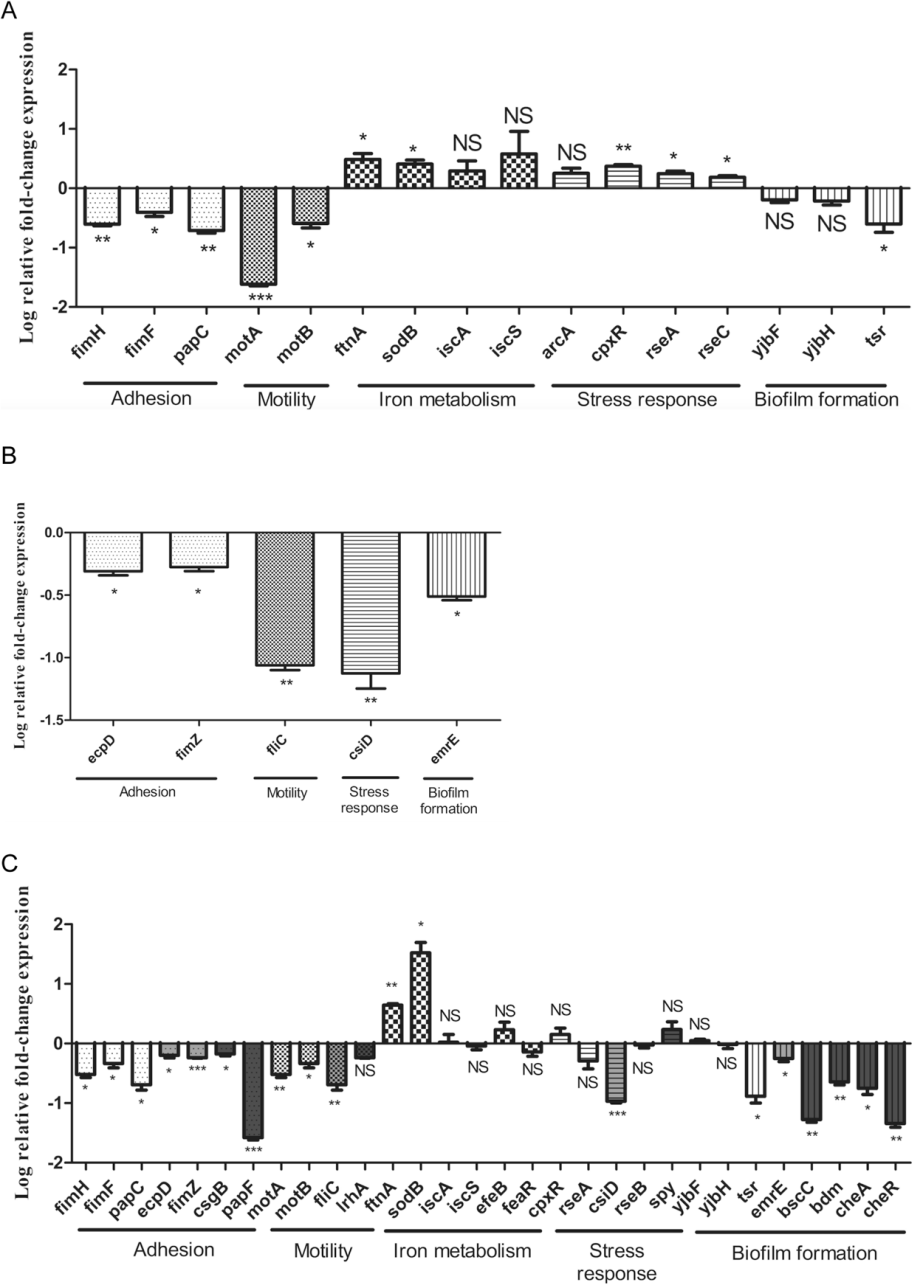
Log relative fold-change in mRNA expression by qRT-PCR of genes involved in adhesion, motility, biofilm formation, iron metabolism and stress response for G50 strain after treatment with cranberry (190 μg/mL) (**A**), propolis (102.4 μg/mL) (**B**) and combined (**C**). The average relative fold-change compared to the control urine/LB condition. The errors bars represent the standard deviation from three different experiments.

For the cranberry and propolis alone, we confirmed the same gene expression changes as those observed with microarray regardless of the metabolic pathway (Fig. 2).

For cranberry + propolis treatment, the majority of results were confirmed although some differences were noted, in particular for genes involved in iron metabolism. Indeed, whilst the majority of genes involved in this pathway were overexpressed (*ftnA, sodB, iscA, efeB, cpxR*), this up-regulation was non-significant for three genes (*iscA, efeB, cpxR*) (Fig. 2). Moreover, two other genes (*iscS* and *feaR*) were down-expressed according to qRT-PCR, although these were also not significant. Also *lrhA* (motility) and *rseB* (stress response) gave opposite, although non-significant results on qRT-PCR compared to microarray. *csiD* is up-expressed according to microarray data upon exposure to propolis alone, but is significantly down-expressed according to qRT-PCR data with propolis alone and cranberry + propolis.

### Propolis potentiates the effect of cranberry on biofilm

**Figure 3:**
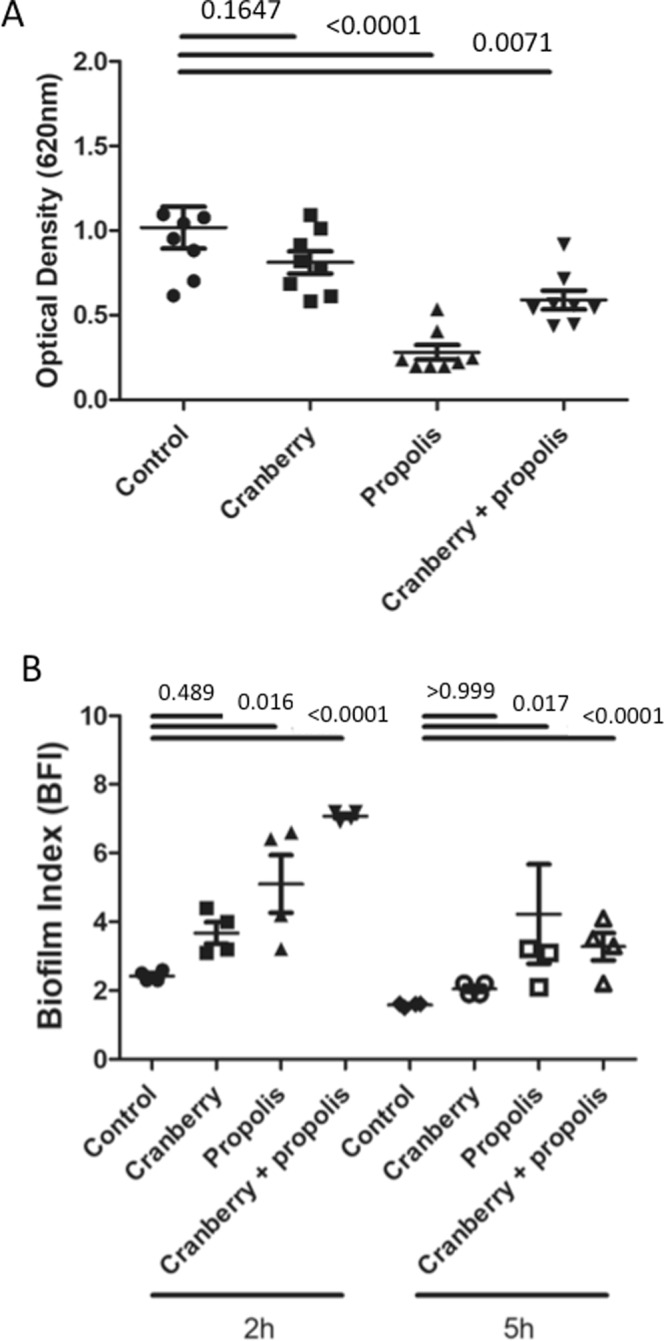
Effect of cranberry, propolis and both on biofilm formation. (**A**) Complete biofilm formation was determined by crystal violet experiment. The optical density (OD) is directly linked to the biofilm formation. (**B**) The kinetics of early stages of biofilm for G50 was determined by the Biofilm Ring Test at 2 h and 5 h. Biofilm Index (BFI) >7 indicates absence of biofilm and BFI < 2 indicates a fully formed biofilm. Means and standard errors for three independent replicate are presented. Statistical differences between different growth conditions at each time were obtained by ANOVA.

Biofilm formation results were confirmed with crystal violet experiments. Complete biofilm formation of untreated *E. coli* G50 was obtained after 48 h (OD620 = 1.02 ± 0.35) (Fig. 3A). Cranberry treatment had no significant effect on the formation of the biofilm (OD620 = 0.81 ± 0.19, p = NS), whereas propolis alone and combined with cranberry significantly inhibited biofilm formation (OD620 = 0.28 ± 0.12, p < 0.001 and 0.59 ± 0.16, p = 0.0071, respectively).

The Biofilm Ring test® was performed to evaluate the effect of cranberry + propolis on the capacity of *E. coli* to form biofilm. Untreated *E. coli* G50 constituted a largely complete biofilm by 2 h and this biofilm was fully formed by 5 h (BFI = 2.43 ± 0.15 at 2 h and 1.58 ± 0.05 at 5 h) (Fig. 3B). Cranberry alone had no significant impact on biofilm formation kinetics, with cranberry-treated cells showing similar BFI values at both timepoints to that of untreated cells (3.68 ± 0.6 at 2 h and 2.05 ± 0.2 at 5 h, p = NS). In contrast, a slowdown of biofilm formation was detected for propolis-treated G50 after 2 h and 5 h (BFI = 5.10 ± 1.7 and 4.22 ± 2.9 respectively, p < 0.05). This effect was significantly increased when G50 was incubated with cranberry + propolis (BFI = 7.08 ± 0.15 at 2 h and 3.28 ± 0.79 at 5 h, p < 0.0001).

**Figure 4:**
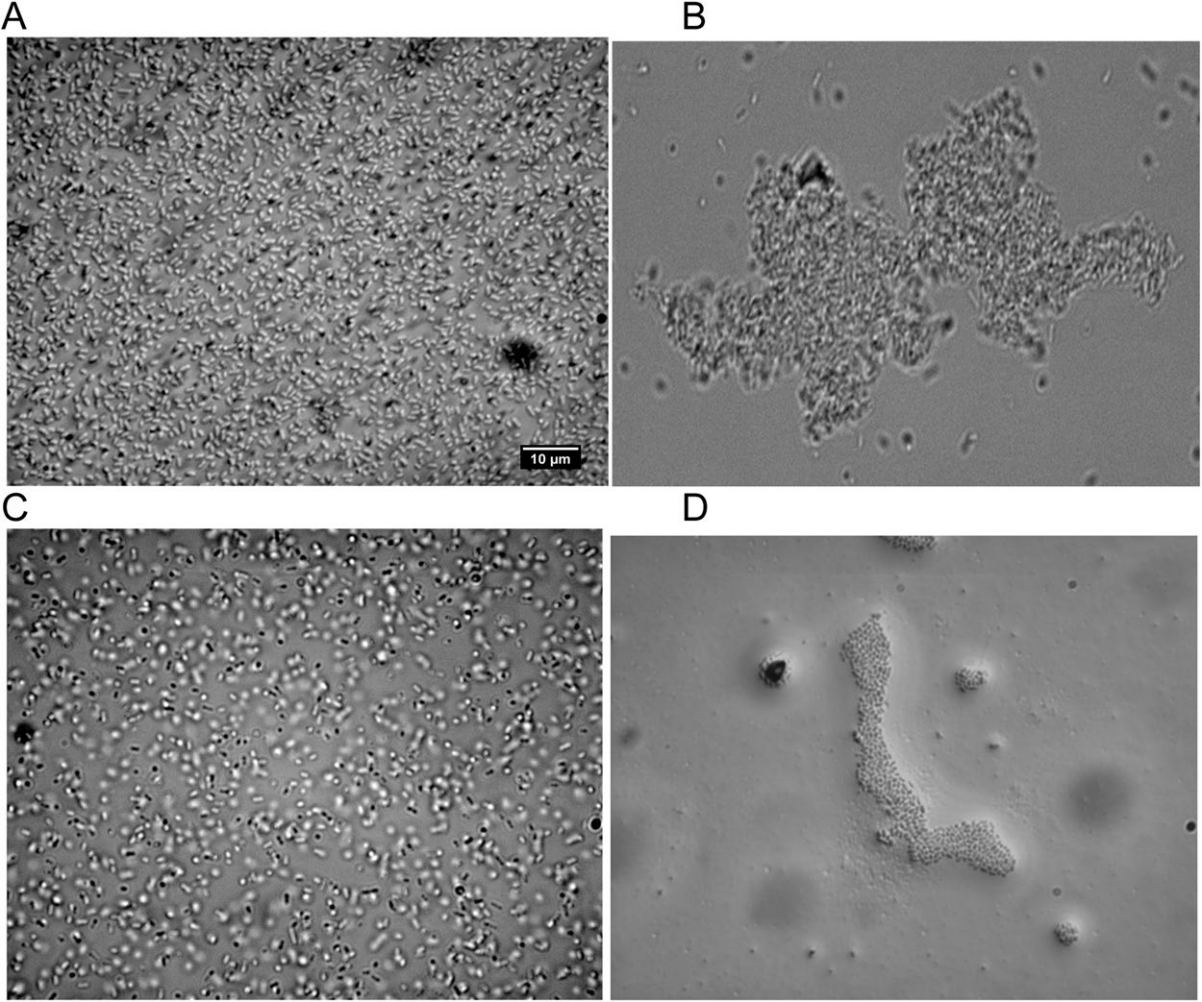
Image of strain G50 immediately obtained in a solution without (**A**), with cranberry (190 μg/mL) (**B**), with propolis (102.4 μ/mL) (**C**) and with cranberry + propolis (**D**) with an inverse microscope.

To further investigate the lack of impact of cranberry on biofilm formation, we observed the bacterial behavior with cranberry alone, propolis alone and both by an inverted microscope. As seen in Fig. 4, we saw an immediate attraction of all the bacteria to each other. This attraction was not due to an active movement from the bacteria but more likely due to an electrostatic movement independent of bacteria. The bacteria formed a mass weaker than a biofilm, as brief agitation of the cells by vortex was sufficient to disperse the bacteria.

### Propolis potentiates the effect of cranberry on motility

To confirm the impact of cranberry and/or propolis on mobility, bacterial swimming and swarming motilities were quantified on soft agar at 48 h.

**Figure 5:**
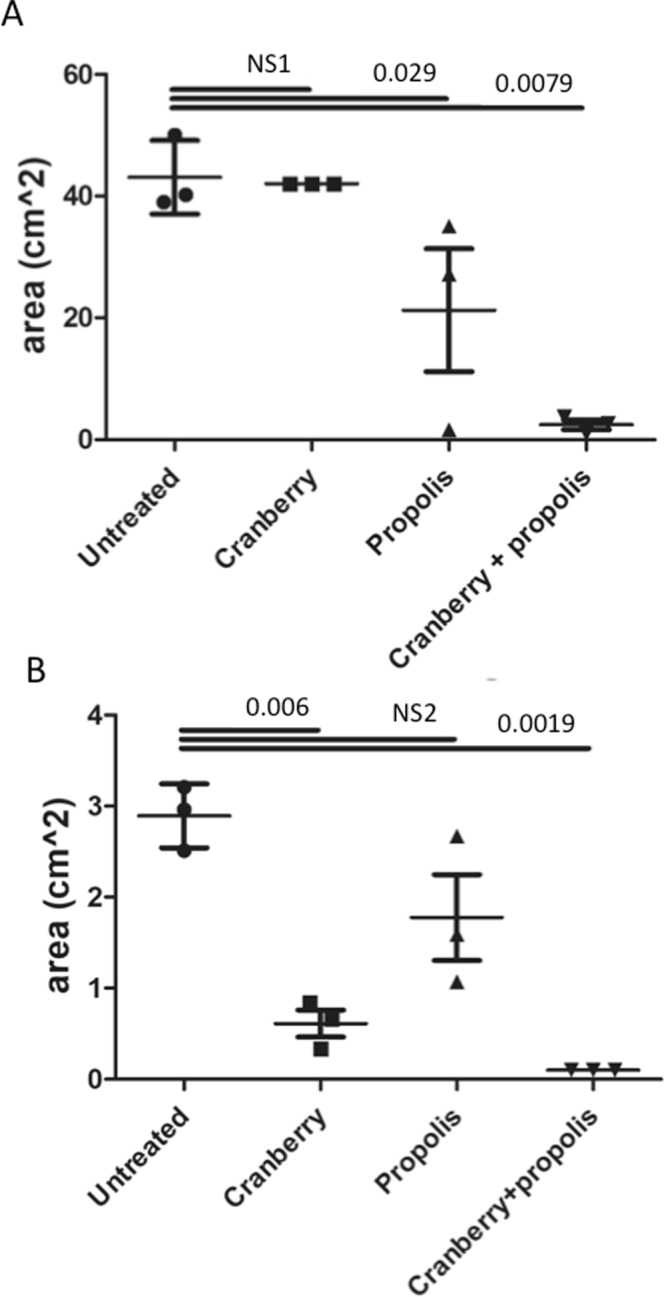
Comparative results of swimming (**A**) and swarming (**B**) assays for G50 in different growing conditions (cranberry, propolis, and combined) at 48 h. The errors bars represent the standard deviation from at least two independent assays. A. NS1, not significant: p > 0.9999; B. NS2: p = 0.2625.

As shown in Fig. 5, cranberry and propolis alone did not affect the swimming behavior although a significant decrease of swimming could be noted with propolis at 48 h (43.1 cm^2^ ± 1.9 for untreated, 42.1 cm^2^ ± 0.5 with cranberry, 21.2 cm^2^ ± 7.5 with propolis; p = 0.029). In contrast, the combined cranberry + propolis considerably affected swimming mobility (2.4 cm^2^ ± 1.9, p < 0.001). Swarming behavior was not affected by propolis alone (2.9 cm^2^ ± 0.5 for the untreated vs 1.9 cm^2^ ± 1.1 with propolis, p = NS) at 48 h, but a significant effect could be noted with cranberry alone and in combination with propolis (0.6 cm^2^ ± 0.4 with cranberry, p < 0.05 and 0.1 cm^2^ ± 0.1 with the combination, p < 0.001).

## Discussion

**Figure 6:**
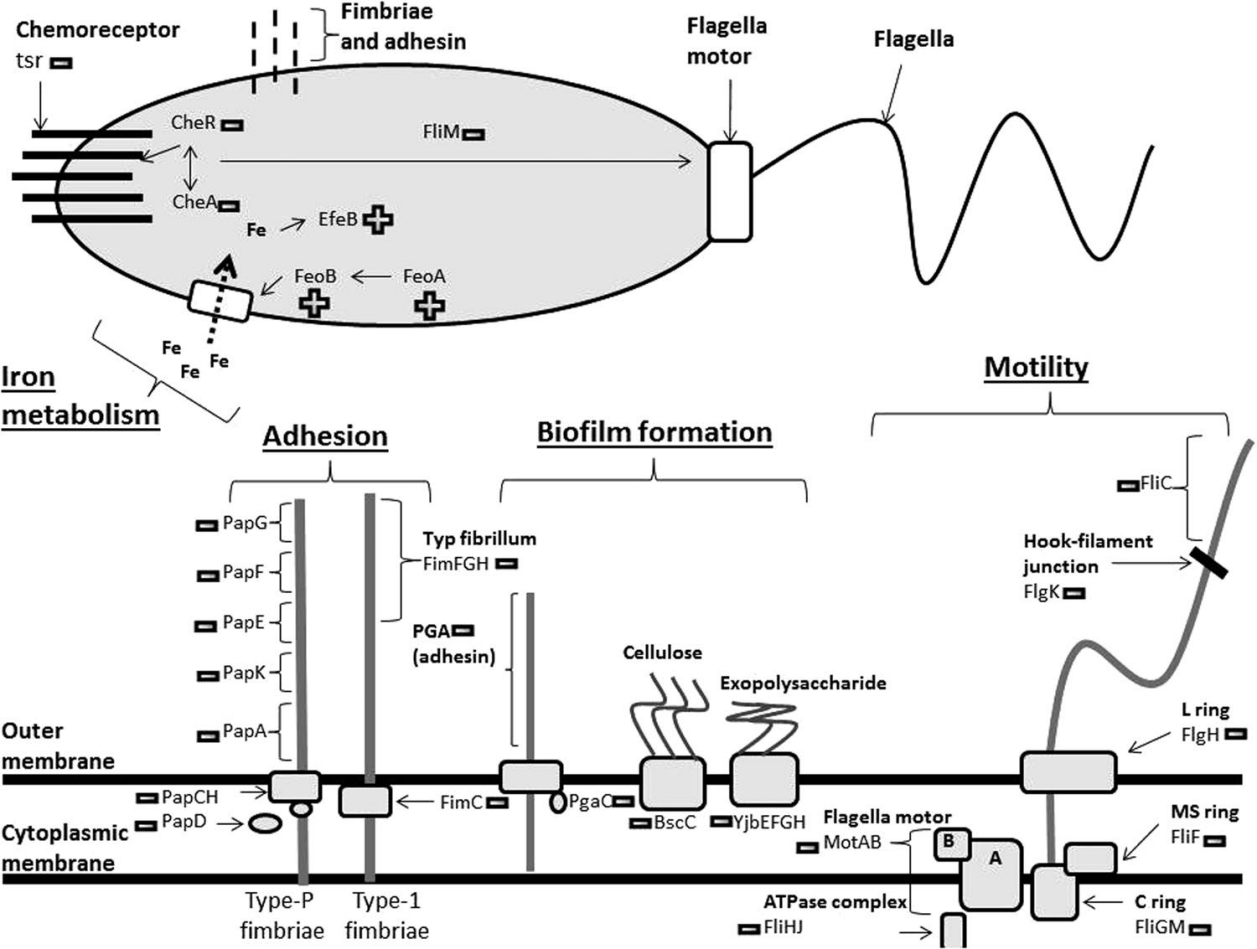
Simplified model for the metabolic pathways impacted by the association of cranberry (*Vaccinium macrocarpon*) and propolis. Genes related to adhesion, motility, iron metabolism and biofilm formation are represented with their variation: + corresponds to an up-regulation; − to a down-regulation.

This study confirms that propolis potentiates the effect of cranberry on transcriptional profiles of UPEC. Previously, we showed that administration of PACs plus propolis offers some protection against bacterial adhesion, bacterial multiplication and virulence in the urinary tract^15^. Here we observed for the first time that more bacterial pathways involved in UPEC pathogenicity (adhesion, motility, biofilm, stress) were affected by the combination of cranberry + propolis than those affected by the two components alone (Fig. 6).

Some studies have already shown that PACs play a role in the modulation of adhesion in UPEC^9,20,23^. By taking a transcriptional approach, we confirmed that the inhibition of UPEC adhesion is not only due to an external modification of the bacterial membrane. This anti-adhesion is mainly the effect of PACs on “strategic” genes involved in *E. coli* adherence (Fig. 6). Thus the two main adhesins (Type-1 and Pap fimbriae)-encoding genes (*fimH* and *pap*) were significantly down-regulated (p < 0.001). Moreover, PACs also affected chaperone-usher fimbriae proteins such as Yad. This protein is important in bacterial adhesion but also in biofilm formation and motility. By down-expressing *yad*, cranberry treatment affected all these downstream functions. Interestingly, in a murine model, *yad* gene knockouts reduced bacteria fitness in bladder and kidney in comparison with wild-type^24^. The same results were observed for *hof*BCQ, Type-IV pili-encoding genes involved in mobility, adhesion, and biofilm formation, conferring an advantage to bacteria colonizing the kidney^25,26^. Our results also showed for the first time that propolis had UPEC anti-adhesion activity, notably on Type-I and Type-P fimbriae-encoding genes (*fim*Z, *pap*A) and *ecpD* (*Escherichia coli* Common Pilus), an adhesion-encoding gene, which contributes to adhesion of human epithelial cells and biofilm development^27,28^. Interestingly, cranberry + propolis treatment increased this anti-adhesion effect, while some other fimbriae-encoding genes (*fimC*, *papF*) were also down-expressed. *csgB* gene was also down-regulated by the combined treatment. This gene encodes a subunit which composes the curli filament, an important factor involved in host cell adhesion, invasion and in biofilm formation^29^.

Other pathways were also affected by these treatments. We observed that cranberry alone caused down-regulation of genes involved in motility pathway such as *motAB*, two essential genes participating in flagellar rotation^30^. Moreover, cranberry exposure led to an up-regulation of *clpXP*, two protease-encoding genes controlling expression of flagellar genes by degradation of mRNA of genes related to flagellar biosynthesis (*flh*CD)^31,32^. Cranberry treatment also resulted in up-regulation of *crl*/*rpo*S genes, which are mostly involved in stress response pathways during stationary and exponential phases. Both of these genes repress genes involved in motility (*fliA*, *flgM*)^33^. Finally, cranberry treatment triggered up-regulation of *nsrR*, a nitric oxide-sensitive repressor of transcription-encoding gene. Previous experiments have shown that over-expression of *nsr*R led to a reduction of bacterial motility in low-agar plates experiments^34^. Interestingly, propolis also had an effect on genes involved in motility by causing a down-expression of *fliC*, encoding a protein of the flagella^35^. Moreover, cranberry + propolis treatment had a significant impact on genes involved in motility, by down-regulating two other genes (*fliA* and *fliR*) participating in flagellar biosynthetis^36^. A key repressor of motility, *lrhA* was also over-expressed under this condition. Knockout of this gene increases bacterial motility and chemotaxis^37^. To corroborate these results, we performed phenotypic experiments of bacterial swimming and swarming. The phenotypic assays confirmed that cranberry + propolis treatment significantly reduced bacteria swimming and swarming.

Secondly, we showed that many genes involved in iron metabolism were up-regulated by cranberry treatment, demonstrating that UPEC adapts to the presence of cranberry PACs by reducing iron storage and up-regulating iron acquisition systems. This confirms that cranberry limits iron availability^21,22^. For example, *fntA* which encodes a major iron storage protein^38^, is up-regulated. Moreover, *feoA* and *B* genes encoding proteins involved in transport of ferrous iron into bacteria^39^, were up-regulated, and their repressor (*feoC*) was down-regulated. Whilst propolis alone did not affect iron metabolism, the combination of cranberry + propolis significantly modified this pathway. Indeed more genes involved in iron transport (*efeB*, *fepA*)^40,41^, and transcriptional activator for iron transporter (*feaR*)^23^, iron storage (*ftnB*)^42^ are up-regulated than conditions with cranberry and propolis alone.

Thirdly, we observed that genes related to stress response were up-regulated in response to cranberry alone. For example, *oxyR*, a gene encoding a key regulator in the defense against hydrogen peroxide (H_2_O_2_), was overexpressed^43^. The ARC two-components system (Anoxic Redox Control) allows facultative anaerobic respiration in response to a modification of nutrients condition. When this system is activated, respiratory metabolism is repressed and fermentative metabolism is promoted^44^. We observed that *arcA*, the gene encoding response regulator protein, was up-regulated in the cranberry-treated bacteria. As aerobic respiration is important for adhesion pathways and virulence of UPEC^45^, we assume that cranberry PACs could also decrease UPEC adhesion and virulence by promoting an anaerobic respiration. Moreover, PACs have previously been shown to impact expression of *rpoS*, a gene involved in stress response (pH, osmotic and oxidative stress) in the exponential phase^32^, and *cpxR*, a gene encoding a part of the CpxRA two-components system which is activated in response to membrane stress^46^. Microscopic observations confirmed that PACs induce an attraction between bacteria, possibly due to a modification of negative charged membranes of the bacteria and of surface hydrophobicity as previously observed^47^. Although propolis had a minor effect on UPEC stress response, cranberry + propolis impacted significantly more stress response genes than cranberry alone. For example, *rseB* gene, which is the sensor of environmental stress^48^ and *spy*, a gene encoding an activator of *cpx*-controlled genes^49^ were up-regulated in the combined treatment.

Finally, we observed that many genes involved in biofilm formation were modified. Most genes of this pathway were down-regulated, as previously noted for different pathogens (*E. coli*, *P. aeruginosa* or *Enterococcus* sp.)^21,47,50–52^, although other studies contradict this^53,54^. Our results confirm that cranberry negatively impacts biofilm formation, by a reduction of exopolysaccharide, adhesin and chemoreceptors production. For example, poly-β-1,6-N-acetyl-D-glucosamine (PGA) is an adhesin which is necessary to maintain biofilm structure, and PgaC is required for the synthesis of this biofilm^55^. We observed that cranberry exposure caused down-regulation of the *pgaC* gene and the *yjbEFGH* operon. This operon is involved in the production of the exopolysaccharide, an important part of extracellular matrix involved in biofilm^56^. Finally *tsr* and *cheA* genes were down-expressed in cranberry alone-treated cells. These genes encode two important chemoreceptors for bacterial communication and biofilm formation^57^. Propolis exposure also has an impact on biofilm formation; *emrE*, an efflux pump-encoding gene was down-regulated in response to propolis treatment. As previously shown, knockout of this gene strongly decreased biofilm formation^58^. However, as we noted for the other pathways, cranberry + propolis had an even greater impact on biofilm formation. In addition to the genes previously described, cranberry + propolis treatment triggered down-regulation of: the *bscABZC* operon (involved in cellulose production)^59^, *bdm* (biofilm-dependent modulation) gene (which is related to osmotic stress biofilm formation)^60^, *tqsA* gene (involved in quorum-sensing) and other genes involved in adhesion and production of matrix (Table S1)^61^. Biofilm formation is based on three major pathways: adhesion, motility and chemotaxis, all of which are negatively impacted by cranberry + propolis exposure. To corroborate these results, we performed two phenotypic experiments on biofilm formation. We confirmed that early biofilm formation and the final biofilm were strongly reduced by cranberry + propolis exposure.

In conclusion, this study highlighted that exposure to cranberry + propolis significantly increased the effect of cranberry on UPEC metabolism by down-regulation of genes involved in adhesion, motility and biofilm formation and by up-regulation of genes involved in stress responses and iron metabolism. In the context of increasing multidrug resistant bacteria and limited new antimicrobial solution, combined treatment could represent an interesting strategy to prevent UTI.

## Methods

### Bacterial strain, microbial culture and preparation of extracts

All the assays were performed with an UPEC strain previously isolated from a patient with cystitis (G50)^62^.

A mix of filtered urine (filtered by a vacuum-driven filtration system with a 0.22 µm membrane, Millipore^©^ (Billerica, Massachusetts, USA)) and Luria-Bertani broth (LB) growth medium (Invitrogen, Villebon sur Yvette) was used to culture bacteria for RNA extraction. Brain-Heart Infusion (BHI) growth medium (CondaLab, Madrid, Spain) was used to grow bacteria for Biofilm Ring-Test^®^ and crystal violet experiments, and LB growth medium culture for microscopy and mobility assays.

Cranberry extract (*V. macrocarpon*) was obtained by dissolving dried cranberry (Exocyan cran BL-DMAC 6% (Nexira, Rouen, France)) in phosphate buffered saline (PBS) and sterilized by filtration. The concentration of PAC-A was measured by BL-DMAC (colorimetric method^63^). Final concentration of PAC-A used was standardized to contain 190 µg/mL. The reconstituted PAC extract was stored at −20 °C in the dark.

The propolis extract (Plantex, Sainte-Geneviève-des-Bois, France) used in this study is a hydroalcoholic extract of blended propolis from various origins mixed 60/40 w/w with carob. The propolis was diluted in 50 mL of PBS and incubated at 37 °C with agitation at 100 rpm for 8 hours. Then, the solution was clarified by centrifugation (4000 rpm, 20 °C, 10 min) and the supernatant sterilized by filtration.

### RNA extraction and cDNA synthesis

G50 isolate was grown in urine and LB broth alone, with cranberry PAC extracts (190 µg/mL), propolis extracts (102.4 µg/mL) and both to an OD600 of ≈ 0.6. Total RNA from bacterial samples was extracted with Tryzol (Invitrogen) according to the manufacturer’s instructions and samples were purified with the RNeasy mini kit (Qiagen, Courtaboeuf, France). All the RNA extraction experiments are performed in triplicate. RNA was treated with the RNase-Free DNase Set (Qiagen). Purity and concentration were determined using the Nanodrop^TM^ 2000 spectrophotometer (Fisher Scientific, Pittsburgh, PA, USA). cDNA was synthesized from 1 μg of total RNA for each sample, using the iScript^TM^ Select cDNA Synthesis kit (Bio-Rad, Hercules, CA) with random primers according to the manufacturer’s instructions.

### Microarray analysis

Five hundred nanograms of total RNA were used for labelling. The one-color microarray-based prokaryote analysis protocol (FairplayIII Microarray labelling, Agilent) was used according to the manufacturer’s instructions to synthetize and label cDNA. Labelled cDNA was purified with Qiagen RNeasy MinElute clean up kit according to the manufacturer’s instructions and quantified using the Nanodrop^TM^ 2000 spectrophotometer. cDNA were hybridized onto slides (AMADID GE, *E. coli* 8 × 15 K, Agilent®). The microarray images were analysed using GenePix software v6 (Axon Instruments). The data were normalized using the robust multiarray average algorithm (RMA)^64^. Feature Extraction software (version 10.7.1.1, Agilent Technologies) was used to obtain raw data and analyze the array images. The data were imported into GeneSpring, software (version 12.5, Agilent Technologies) to complete the analysis. A Lowess curve (locally weighted linear regression curve) was fitted to the plot of log intensity versus log ratio, and 40% of the data were used to calculate the Lowess fit at each point. The curve was used to adjust the control value for each measurement. If the control channel signal was below a threshold value of 10, then 10 was used instead. For each condition, data set a list of genes was prepared showing at least 2-fold differential expression levels between untreated and treated conditions by using Student’s *t*-test and applying the Benjamini and Hochberg false discovery rate (multiple testing correction, MTC) test with a *p* value cut off of 0.05.

### Comparative real-time qRT-PCR

To confirm the results found in microarray analysis, transcript levels analysis was performed by quantitative reverse transcription qRT-PCR using randomly selected genes involved in the main metabolic pathways of *E. coli* (Table S1). Real-time PCR assays were performed in a LightCycler®480 device using the LightCycler FastStart DNA Master^Plus^ SYBRGreen I kit with 100 ng of cDNA and 10 pmol of target primers (Table S1). The specificity of the PCR products was tested by melting-point analysis. Amplifications were performed in duplicate from three different RNA preparations. The 2^−*ΔΔCT*^ method was used to analyze transcriptional changes in target genes using *gapdh* as the housekeeping control gene. Data were log transformed to obtain a fold change difference between the different studied conditions^65,66^.

### Measure of constituted biofilm by crystal violet

Biofilm development was also assessed by incubating bacterial cultures after an overnight incubation (37 °C, 150 rpm). The culture was diluted 1000-fold in BHI to obtain a final optical density at OD600 ≈1, and 200 μL aliquots were loaded into wells of a 96-well polystyrene microtiter plate (BD Falcon, USA). The plates were incubated at 37 °C for 24 h and 48 h under stationary conditions to allow biofilm formation. The adherent biomass was quantified using the crystal violet assay. Thus, after incubation, the wells were gently washed twice with sterile PBS (pH 7.0) to remove non-adherent cells, planktonic cells. After air drying (10 min), the unattached cells were fixed with 200 μL of 99% ethanol for 30 min. 200 μL of a 0.1% crystal violet solution was added to each well to stain the cells (30 min). Plates were then rinsed three times to remove any unattached crystal violet and air dried. The crystal violet in the stained biofilm was then dissolved in 33% acetic acid solution. The absorbance at OD620 was measured to estimate the biofilms that were formed^53^. Each experiment was repeated twice with three technical replicates.

### Kinetics of biofilm formation

Kinetics of early biofilm formation was explored using the Biofilm Ring Test® (BioFilm Control, Saint Beauzire, France) as previously described^67^. Briefly, 200 μL/well of standardized bacterial cultures were incubated at 37 °C in 96-well microtiter plates. The test was performed using toner solution TON004 in the presence of magnetic beads 1% (v/v) mixed in BHI. At different time points (0, 2 and 5 h) without shaking (static culture), wells were covered with a few drops of contrast liquid (inert opaque oil). Then, plates were placed for 1 min onto a magnetic block carrying 96 mini-magnets and scanned with a specifically designed plate reader (Epson scanner modified for microplate reading). The images of each well before and after magnetic attraction were analysed with the BioFilm Control software generating a BioFilm Index (BFI) reflecting the adhesion strength of the strain in the different conditions. A high BFI value (>7) indicates a high mobility of beads under magnet action, corresponding to an absence of biofilm formation, while a low value (<2) corresponds to a complete immobilization of beads due to sessile cells. Two independent experiments with at least two repeats were performed per condition (cranberry with/without propolis) and per incubation time.

### Motility assays

The motility of the G50 strain in different conditions was evaluated using soft LB-agar plates as described previously: swim plates containing 0.25% of agar and swarm plates containing 0.5% of agar supplemented with 0.5% of glucose^68^. All plates were allowed to dry overnight at room temperature before use. Briefly, bacteria grown overnight in LB were diluted 1000-fold in LB and incubated at 37 °C until stationary phase (to an OD600 of ≈ 0.7). Swarm plates were inoculated into the middle of soft agar surface by spotting with 5 µl of standardized culture. Swimming plates were seeded with the same inoculum below the agar surface using a sterile inoculating needle. Plates were incubated for 48 h at 37 °C. The diameter of the migration zones produced by the strain at different conditions was measured, recorded and calculated using Image J software. Swimming and swarming experiments were performed independently three times.

### Optical microscopy

A bacterial solution grown in LB alone, with cranberry PAC extracts (190 µg/mL), propolis extracts and both was prepared to an OD600 of ≈ 0.1. The suspension was spread uniformly over a glass slide and immediately observed on an inverted microscope (Leica). The different preparations were also observed after vortex of the solution.

### Statistical analysis

Statistics and graphs were prepared using the software package GraphPad Prism 6.0. The effects of cranberry and/or propolis on the expression of selected genes and motility were assessed using one-way ANOVA followed by Dunnett’s multiple comparisons test. Log-transformed data were used for real-time RT-PCR. Kinetics of biofilm formation were compared with a two-way ANOVA followed by Dunnett’s multiple comparisons test. The crystal violet experiments were assessed using a Student’s t-test. p < 0.05 was considered to reflect a statistically significant difference.

## Electronic supplementary material

Table S1
